# Genetic sexing of subadult skeletal remains

**DOI:** 10.1038/s41598-023-47836-9

**Published:** 2023-11-22

**Authors:** Irena Zupanič Pajnič, Teo Mlinšek, Tadej Počivavšek, Tamara Leskovar

**Affiliations:** 1 Institute of Forensic Medicine, Faculty of Medicine, University of Ljubljana, Korytkova 2, 1000 Ljubljana, Slovenia; 2 Centre for Interdisciplinary Research in Archaeology, Department of Archaeology, Faculty of Arts, University of Ljubljana, Ljubljana, Slovenia

**Keywords:** Genetics research, Genetic markers

## Abstract

When subadult skeletons need to be identified, biological sex diagnosis is one of the first steps in the identification process. Sex assessment of subadults using morphological features is unreliable, and molecular genetic methods were applied in this study. Eighty-three ancient skeletons were used as models for poorly preserved DNA. Three sex-informative markers on the Y and X chromosome were used for sex identification: a qPCR test using the PowerQuant Y target included in PowerQuant System (Promega), the amelogenin test included in ESI 17 Fast STR kit (Promega), and a Y-STR amplification test using the PowerPlex Y-23 kit (Promega). Sex was successfully determined in all but five skeletons. Successful PowerQuant Y-target, Y-amelogenin, and Y-chromosomal STR amplifications proved the presence of male DNA in 35 skeletons, and in 43 subadults female sex was established. No match was found between the genetic profiles of subadult skeletons, and the elimination database and negative control samples produced no profiles, indicating no contamination issue. Our study shows that genetic sex identification is a very successful approach for biological sexing of subadult skeletons whose sex cannot be assessed by anthropological methods. The results of this study are applicable for badly preserved subadult skeletons from routine forensic casework.

## Introduction

When subadult skeletons are recovered in forensic casework, they need to be identified^1^, and genetic sexing is one of the first steps in the identification of unknown skeletal remains. In ancient analyses, sex is one of the most important parts in establishing the biological profile of an individual, which is used in archaeology to better understand past human societies, their social stratification, demography, gender roles, and burial practices^2^. Despite the apparent simplicity of limited options (male or female), sex assessment using macroscopic methods based on morphological features can be unreliable, especially when dealing with adult individuals with ambiguous sex characteristics, and/or poorly or partially preserved remains^3^. When dealing with subadults, morphological methods for sex assessment do not achieve the same level of accuracy as with adults^4^ and are currently not advisable^5,6^, and molecular genetic methods for sex identification can help in solving the problems of subadult skeletons with morphometrically undetermined sex. Some studies performed on adult skeletons have indicated that DNA testing has strong potential to improve the accuracy of assessing morphological sex^7–12^, and in this study sex identification was performed on subadult skeletons using molecular genetic analyses.

Females and males genetically differ in sex chromosomes, females having two X chromosomes and males having one X and one Y chromosome. Genetic sexing uses this most obvious difference between the two sexes, and DNA targets on the X and Y chromosome are amplified in polymerase chain reaction (PCR). Different sex-informative markers on the X and Y chromosome are used in routine forensic genetic analyses, and they can be implemented in sex assessment of aged skeletons, especially subadults, where it is not anthropologically possible to determine sex with high accuracy. Some forensic sex-informative tests amplify the targets on the X and Y chromosome (amelogenin test), and some of them only the targets on the Y chromosome; for example, the qPCR Y-target amplification test and Y chromosome short tandem repeat (STR) amplification test^13^. The cheapest and most rapid is the qPCR Y-target amplification test, which is part of some commercially available forensic qPCR kits that amplify not only autosomal targets (usually two autosomal targets are included—short and long—for DNA degradation assessment), but also Y-chromosomal targets for quantification of male DNA. For instance, in the PowerQuant System (Promega, Madison, WI, USA), two multicopy loci are amplified on the Y chromosome and amplicons of 81 bp and 136 bp are produced^14^. In the Human Quantifiler Trio Kit (Thermo Fisher Scientific, TFS, Wathman, MA, USA), multiple copies on the Y chromosome are targeted and amplicons of 75 bp are amplified^15^. In the Investigator Quantiplex Pro RGQ kit (Qiagen, Hilden, Germany), two Y-chromosomal targets are analyzed targeting the same locus, and amplicons of 81 bp and 359 bp are generated^16^. The amelogenin test, which amplifies the targets on the X and Y chromosome, is one of the oldest DNA sex tests^17^. Among the homologous genes of the X and Y chromosome, the amelogenin gene is the most suitable for genetic sexing because the very short target of intron 1 is amplified and generates products approximately 100 bp long that differ in length between the X and Y chromosome^18^. In intron 1, a 6 bp deletion occurred on the X chromosome during its evolution, and so the amplicon of the X chromosome is shorter than the amplicon of the Y chromosome, and when amplifying female DNA (XX) products of the same length are produced, and in male DNA (XY) products of different lengths (differing by 6 bp) are generated^19^. Because of the short products, the amelogenin test is also suitable for determining the sex of highly degraded DNA^20^. The amelogenin test is included in practically all commercial human identification kits, which allows genetic sexing in addition to determining identity^17^. The third forensic sex-informative test, which is very often used in analysis of sexual assault cases^21,22^, is the Y-STR amplification test and, because of the different length of amplicons produced on STR loci, partial profiles are expected in badly preserved samples with high degradation of DNA.

Because ancient DNA (aDNA) is of low quantity and quality^23–26^, high degradation limits the success of PCR amplification, especially when longer DNA targets are used for analyses^27^. Because the qPCR Y targets and amelogenin targets included in autosomal STR typing kits are much shorter than STR targets on the Y chromosome, they are more promising for genetic sexing of aDNA. In addition to low DNA yield and high degradation, the success of aDNA analysis is also potentially limited by inhibitors and possible contamination^28,29^, and this requires that precautions be followed to avoid and identify contamination by modern DNA together with verification of aDNA authenticity^30–32^.

In this study, 83 subadult skeletons for which sex assessment was not possible through anthropological analysis were genetically examined on different X- and Y-chromosomal markers. All individuals studied were buried in a modern-era cemetery (16th to late 19th century) in Ljubljana and recently excavated. Because skeletons are difficult to obtain in forensics for research purposes, ancient skeletons were used as models for highly decomposed and skeletonized subadult forensic skeletal remains. Petrous bones—which among all the bones of the human body generate the highest DNA yields in ancient^33,34^ and badly preserved forensic skeletal remains^35^—were sampled from almost all subadults. Better preservation of DNA in the petrous bone is related to high density^33^, absence of remodeling (Pinhasi et al.^41^), and a high concentration of osteocytes^36,37^.

This research was approved by the Medical Ethics Committee of the Republic of Slovenia (0120–256/2022/3), and informed consent was supplied by the individuals entered in the elimination database. Responsibility for archiving the site is entrusted to Ljubljana Museum and Galleries (MGML), whose curator Martin Horvat agreed with this investigation.

## Materials and methods

### Description of the Ljubljana–Polje archeological site and selection of bone samples

**Figure 1:**
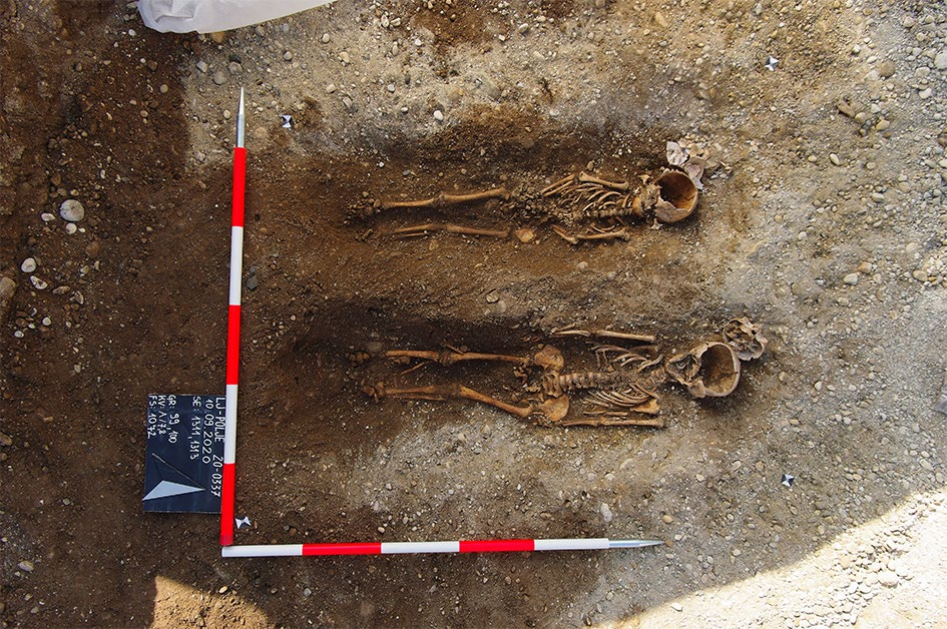
Skeleton 1311 from grave 99 and skeleton 1313 from grave 100.

Prior to infrastructure work in the Polje neighborhood in the eastern part of Ljubljana, Slovenia, archeological excavations uncovered the northern part of a modern-era cemetery. An archeological excavation took place in August and September 2020, when 216 graves were documented. According to archival sources, the cemetery belongs to Assumption Church in Polje, which was first mentioned in 1325, and the cemetery was active between the early 16th and late 19th centuries^38,39^. In the early stages of the cemetery, clearly separated graves of adults predominate. Later, there was probably a lack of space and newer graves began to cut into the older ones. Some of the graves were even reused because disarticulated remains were discovered at the edge of the burial pits, and a new individual was placed in the center. Based on the archeological findings, it was determined that the individuals were buried clothed in wooden coffins. To establish biological profiles, anthropological analyses were performed on all the skeletal remains excavated from the Ljubljana–Polje archaeological site, and 29% of the individuals were adults 18–60 or more years old, and 71% were non-adults up to 17 years old. Due to the state of preservation, macroscopic assessment of sex was only possible for 75% of the adults, of whom 54% were female and 21% male. The sex of subadults was not assessed because macroscopic methods are not reliable^40^. Based on the completeness and state of preservation of the skeletal remains, 83 subadults were selected for the research. Most of them had preserved petrous bones, which are the richest source of DNA in ancient skeletons^34,41^, and they were collected for DNA analyses of sex identification. The petrous bone contains organs of hearing and equilibrium. The vestibule, three semicircular canals, and the cochlea form the osseous labyrinth, which is the densest and hardest bone in the body of mammals^42^. Femurs were sampled only in three cases in which petrous bones were not preserved (see Supplementary Material 1, SM 1). The skeletons analyzed differed in their age: 18 skeletons that most likely belonged to stillborn babies or premature babies that died after birth 30 to 40 weeks old in utero, 14 skeletons of newborns up to 1 year old, 23 skeletons from 1 to 6 years old (two skeletons are shown on Fig. 1), and 28 skeletons from 6 to 13 years old. Following anthropological examination, the bones selected were tagged with the names and numbers of the respective graves and skeletons.

We considered intra-bone variability in DNA content when sampling the petrous bones and femurs in order to obtain the bone powder and extract the DNA. The study used the dense part of the petrous bone inside the otic capsule^34^ and the diaphysis from femurs^13,43^. Pinhasi’s method^41^ was used to detach the cochlea from the petrous bone with a sterilized diamond saw (Schick, Schemmerhofen, Germany). In femurs sampled from two subadults up to 2 years old, the two epiphyses were cut away from the diaphysis and were not used for extraction. In a subadult 12 to 13 years old, the femur’s diaphysis was sampled below the greater trochanter.

### Extraction of DNA and measures to prevent contamination

A sterilized diamond saw (Schick) was used to notch femur samples and petrous bones into squares that measured about 5 × 5 mm to facilitate grinding. All of the bones were chemically cleaned and UV irradiated to remove any surface contamination^44^. Chemical cleaning was carried out with 5% Alconox (Sigma-Aldrich, St. Louis, MO, USA), sterile bi-distilled water (Sartorius-Stedim Biotech, Göttingen, Germany), and a solution of 80% ethanol (Merck, Kenilworth, NJ, USA). The bones were allowed to dry before they were cut with a sterilized diamond saw (Schick). Immediately before they were cut, we cooled the bones with liquid nitrogen. Before they were ground, the samples were held under UV light for 30 min using BLX-Multichannel BioLink DNA Crosslinker (Vilber, Collégien, France). All of the tools used for cutting, drilling, and grinding the bones were cleaned with 6% sodium hypochlorite, sterile bi-distillated water, and a solution of 80% ethanol, and this was followed by sterilization in a Europa B xp sterilizer (Tecno-Gaz, Parma, Italy) with incubation at 134 °C for 45 min plus 30 min of UV irradiation using BLX-Multichannel BioLink DNA Crosslinker (Vilber).

Special measures^30, 45^ were followed to prevent contemporary DNA from contaminating the samples, and all procedures for bone extraction were fully separated from the post-extraction procedures^44^. Sample preparation was conducted in a specially designed room for working on old skeletal remains inside a closed MC 3 microbiological safety cabinet (Iskra Pio, Šentjernej, Slovenia). The room was equipped with UV light and a HEPA filter. To obtain the 0.5 g of bone powder needed for DNA extraction, bone samples prepared in advance were ground into fine homogenous powder with a homogenizer (Bead Beater MillMix 20, Tehtnica, Domel, Železniki, Slovenia). As previously described by Zupanič Pajnič^44^ the DNA samples extracted from the bones were cleaned, ground, decalcified, and purified. To eliminate contamination, all the reagents used (except for those labeled DNA-free or DNase-free), the laboratory plastic, water, bleach, and ethanol were sterilized (autoclaving) and exposed to UV irradiation for 30 min with BLX-Multichannel BioLink DNA Crosslinker (Vilber) before they were used. For control of contamination, to each batch of samples extraction-negative controls (ENC) were added (15 extraction-negative controls were processed altogether). The purpose of these was to monitor the purity of the various materials used (reagents and plastics) throughout the entire process^46^. To additionally control for potential contamination, all persons involved were sampled in order to create an elimination database (this included persons involved in analyzing DNA, in anthropological analyses, and in excavations). Saliva was collected with sterile cotton swabs from everyone involved to create the elimination database, and then DNA was extracted from buccal smears. Two different devices were used to purify the elimination database samples and bone samples to prevent ancient samples from being contaminated. A BioRobot EZ1 machine (Qiagen) was used to purify DNA from buccal swabs, and bone sample purification was carried out with the EZ1 Advanced XL machine (Qiagen). Our laboratory uses this device exclusively to purify ancient DNA samples. Instructions provided by the manufacturer^47^ were followed for using the EZ1 DNA Investigator kit (Qiagen) for processing buccal swabs, and this was also in line with the previously published protocol^44^ for processing bone samples.

Three sex-informative tests were applied to perform sex identification. The first one was a qPCR test using the PowerQuant System (Promega), the second one was a Y-STR amplification test using the PowerPlex Y-23 kit (Promega), and the third one was the amelogenin test included in the autosomal STR typing kit ESI 17 Fast (Promega).

### DNA quantification (qPCR Y-target test)

To determine the concentration of DNA and its degradation in bone extracts, qPCR analyses were performed amplifying short (Auto target) and long (Deg target) autosomal targets using the PowerQuant System (Promega). DNA quantity was determined from Auto target measures, and the degradation index was calculated from the Auto/Deg ratio. We used an internal PCR control (IPC shift) to detect any possible presence of PCR inhibitors. The Y-chromosomal target included in the same kit was used to detect the presence of male DNA and served as a first sex informative test for genetic sexing of all ancient subadult skeletons analyzed. Analyses were performed following the technical manual^14^ with the QuantStudio 5 Real-Time PCR system, the PowerQuant Analysis Tool (https://worldwide.promega.com/resources/tools/powerquant-analysis-tool/), and Quant-Studio Design and Analysis Software 1. 5. 1 (Applied Biosystems, AB, Foster City, CA, USA).

The threshold for the Auto/Deg ratio was set at 2 and the IPC shift value was set at 0.30, as recommended by the manufacturer^14^. Laboratory plastic and reagent cleanliness was verified through analysis of negative template controls and extraction-negative controls. Bone extracts, positive controls, and negative controls were quantified in duplicate, following the manufacturer’s recommendations^14^. For all of the bone samples, the DNA quantity obtained from 1 g of bone was calculated on the basis of the Auto target, and this was expressed in ng DNA/g of bone. We multiplied the Auto target results by 100 because 0.5 g of bone powder was used and elution of the DNA took place in 50 μl of TE buffer.

### Y-chromosomal (Y-STR amplification test) and autosomal STR typing (amelogenin test)

The Y-chromosomal STR typing kit PowerPlex Y-23 System (Promega) was used as a second sex informative test, and the autosomal STR typing kit PowerPlex ESI 17 Fast System (Promega) including an amelogenin test as a third sex informative test. PCR was performed following the recommendations of the manufacturer^48,49^, using the Nexus MasterCycler (Eppendorf, Hamburg, Germany). Amplification included both positive and negative PCR controls. Variation in the final volume of bone extracts used in the PCR reaction was based on the quantification results of the PowerQuant Auto target. If the quantification was 0.06 ng per μl of extract or higher, 1 ng of DNA was used as a template. The maximum DNA extract volume (17.5 μl) was used if the quantification was lower than 0.06 ng per μl of extract. Maximum volume was also used for amplification of ENCs. Genetic profiles for the samples were acquired using the SeqStudio Genetic Analyzer for HID (Thermo Fisher Scientific, TFS) combined with the WEN Internal Lane Standard 500 (Promega), SeqStudio Data Collection Software v 1.2.1 (TFS), and GeneMapper ID-X Software v 1.6 (TFS).

The PowerQuant qPCR test was performed in duplicate on all bone samples analyzed and all ENC samples. Based on the qPCR results, the bone samples were divided into low-template DNA samples with a quantity below 0.006 ng of DNA per μl of extract and samples with a DNA quantity above this value. When the maximum possible volume of DNA extract is used for PowerPlex Y-23 and ESI 17 kit PCR amplification, less than 100 pg is amplified if the quantity of DNA is below 0.006 ng of DNA per μl of extract. Amplification of less than 100 pg of DNA can lead to allelic drop-outs due to stochastic effects, and only partial or even no profiles can be expected^50^. Accordingly, all low-template bone DNA extracts were analyzed using the Y-STR amplification test and amelogenin test (see SM1; five samples labeled in blue). The Y-STR amplification test was also performed on 56 bone extracts (see SM 1), in which the qPCR Y target was detected (even if the measures were below the detection limit of the PowerQuant kit, which is 0.0005 ng^51^ and was set in developmental validation for the PowerQuant System), and an additional amelogenin test was performed on those bone samples that produced partial Y-STR haplotypes consisting of fewer than 16 Y-STRs to confirm male sex of skeletons (see SM 1, SM 3, and SM 4, samples labeled in yellow). A high-quality sample from skeleton 1223 with no Y qPCR target detected (see SM 1) was also typed for Y-STRs to confirm no presence of male DNA. Altogether, 60 bone samples were typed for Y-STRs.

The absence of Y qPCR amplicons in bone extract that yielded more than 0.006 ng of DNA per μl of extract indicates female sex of the skeleton. However, to additionally confirm female sex using an amelogenin sex test, eleven subadult skeletons with different ages were selected (see SM 1 and SM 3, samples labeled in gray) and analyzed using the ESI 17 autosomal STR kit. Not all of the bone samples with high DNA yields and no qPCR Y-target detection were analyzed with the amelogenin test to reduce the costs of the reagents.

All ENC samples employed to monitor possible contamination with modern DNA were analyzed using the autosomal ESI 17 STR amplification kit, and Y-STR amplification was performed for ENC samples that produced PowerQuant Y-target amplicons (three out of 15 ENCs; see SM 2, labeled in green).

In addition, all samples included in the elimination database were typed for autosomal STRs, and genetic profiles were compared to those obtained from bone samples to check the authenticity of isolated DNA and to exclude modern DNA contamination of the endogenous bone DNA through differentiation of genetic profiles of the bone compared to profiles in the elimination database. To increase the number of bone samples tested on autosomal STRs and compared to the elimination database, an additional seven male subadults were typed using the ESI 17 kit (see SM 1 and SM 3, samples labeled in orange).

### Ethics approval

The research was performed in accordance with the Declaration of Helsinki and was approved by the Slovenian Medical Ethics Committee (0120-256/2022/3). The individuals included in the elimination database gave their written informed consent.

## Results

### DNA quantification

SM 1 presents bone sample characteristics and summarizes the results for DNA quality and quantity (Auto/Deg ratio, IPC shift, Auto, Deg, and Y target; these last three values are presented as ng DNA/µl of extract), which were acquired by using the PowerQuant System (Promega). In addition to this, the amount of DNA obtained from 1 g of bone is expressed in ng DNA/g of bone. Because duplicate analyses were performed, the average measurements are presented.

More than 0.5 pg of human DNA per μl of extract was detected in all of the bone samples (see SM 1). There was degradation between samples at various levels, ranging from severely to slightly degraded. The range of the degradation index was 0.71–255, and in all except one of the bone samples it surpassed the threshold of 2. Indeed, the sample with the lowest degradation index was the bone sample with the lowest DNA yield, at a concentration of 0.5 pg per μl. This measure is at the detection limit of the PowerQuant kit, meaning that the calculated degradation index may not reflect reliable DNA degradation and degradation could be much higher. In two cases it was impossible to ascertain the degradation value because the Deg target could not be detected. An IPC shift value of more than 0.3 indicates that inhibitors are present; in our case, this occurred in 1 of 83 extracts (see SM 1, labeled in red), indicating high purification efficiency of the magnetic bead technology used in the EZ1 DNA Investigator kit (Qiagen). In skeleton 1640, the IPC shift value exceeded the 0.3 threshold (the IPC shift value was 2.5; see SM 1, labeled in red). However, an almost full Y-STR haplotype was obtained from that skeleton, showing no inhibition of amplification.

### Sex identification

The Y PowerQuant target amplification product was identified in 56 samples (see SM 1), and the quantity of male DNA was very low (mostly below the detection limit of the PowerQuant kit) in 21 of them (see SM 1, labeled in green). All samples with detected PowerQuant Y-target amplicons were typed for Y-STRs, and only 36 of them produced Y-STR haplotypes using the PowerPlex Y-23 kit from Promega (see SM 1 and SM 4). Among the 36 samples with a successful Y-STR typing outcome, only one sample (skeleton 1335) yielded a Y-target measure under the detection limit of the PowerQuant kit, and indeed only four Y-STR loci were successfully amplified (see SM 1). To check whether this Y-STR amplification outcome was due to a minor contamination issue, the ESI 17 Fast autosomal STR kit (Promega), including an amelogenin test, was used for typing, and no STR genotype and no amplicons on the amelogenin gene were obtained, indicating that for skeleton 1335 sex identification is not possible (see SM 1). The sample from skeleton 1335 yielded very low amounts of DNA, and 57 pg of DNA was amplified in the autosomal and Y-chromosomal STR kits. In the other 35 samples with high Y-target DNA yields, 27 samples produced Y-STR haplotypes with more than 16 STRs amplified (see SM 1 and SM 4), and we concluded that they belonged to male individuals. For the remaining eight samples (for which five to 15 Y-STRs were successfully amplified; see SM 1 and SM 4), additional analyses for sex identification were performed using the amelogenin test. Because all of them generated products on the X and Y amelogenin gene, male sex was determined for them (see SM 1 and SM 3). Altogether, 35 skeletons were attributed to males. For skeleton 1299, a low-template DNA extract was obtained, but despite detection of low amounts of the Y target, no Y-STRs were generated and the amelogenin test also failed; accordingly, sex identification was not possible (see SM 1).

For 20 samples with low Y-target measures and high measures of the Auto target and no Y-STR amplification success, female sex was identified (see SM 1). It is possible to explain detection of a low quantity of the PowerQuant Y target in those samples by observing that in three ENCs the qPCR Y target also yielded a product (cf. SM 2, labeled in green), but ENC samples did not produce Y-STR haplotypes or amelogenin/STR profiles, which indicates that qPCR analysis has greater sensitivity for detecting minimal contamination issues. However, because the amounts of non-bone DNA are too low, this does not affect the detection of contamination alleles in Y-STR and autosomal STR profiles.

Female sex was attributed to an additional 23 samples with high measures of Auto target and no Y-target detection, and for 11 of them female sex was also confirmed through an amelogenin sex test that produced amplicons only for the X chromosome (see SM 3, samples labeled in gray). Altogether, female sex was determined for 43 subadult skeletons studied.

For skeletons 1032, 1272, and 1507, low-quantity DNA was extracted and the Y qPCR target was not amplified, Y-STR typing failed, and low-quality partial autosomal STR profiles were obtained with an amplified amelogenin locus (X,Y for skeleton 1032 and X,X for skeleton 1507) or unamplified amelogenin locus (skeleton 1272), resulting in the decision that it was not possible to determine the sex for them (see SM 1, labeled in blue). To summarize, when low concentrations of DNA were extracted from subadults, some tests failed and genetic sexing was not possible for five skeletons analyzed (1032, 1272, 1507, 1299, and 1335; see SM 1 and SM 3, labeled in blue).

Considering the 15 ENCs employed, no Auto or Deg PowerQuant targets were amplified, except in six ENC samples (see SM 2) in which the qPCR product was mostly at the detection limit of the PowerQuant kit or lower, but no autosomal STR profiles were produced (see SM 2). The Y-PowerQuant target was amplified on three ENCs with a quantity lower than 0.0002 ng per μl (see SM 2, labeled in green), and after Y-STR typing no haplotypes were obtained (see SM 2). Comparison of genetic profiles between the elimination database and bones was performed on all bone samples that were typed for autosomal STRs and produced good-quality genetic profiles. In 23 bone samples that generated more than 14 STRs, no match was found in the elimination database. High degradation indexes, combined with pure negative controls and no matches in the elimination database, show that the DNA obtained from ancient bones and the genetic profiles created were authentic to them. The extraction-negative controls did not generate any genetic profiles, which confirms an absence of amplifiable contaminated DNA. Together with no matches found in the elimination database, this indicates that the DNA obtained from the subadult skeletons analyzed can be considered endogenous bone DNA.

## Discussion

To achieve correct sex identification of subadult skeletons, up to three different sex informative tests were combined, and in all but five samples sex identification was successful. All five samples with unsuccessful sex identification yielded low DNA quantities. In low-template DNA samples, male sex could be confirmed only if all three tests were positive for the presence of Y-specific genetic markers. If not, it is also not possible to confirm female sex because of the possibility of drop-outs due to stochastic effects^50^.

Emphasis must be placed on some limitations of the sex informative tests used for sexing the subadult skeletons studied. In the qPCR test, because of lower sensitivity of Y-target amplification in relation to autosomal target amplification (due to higher susceptibility of the Y chromosome to degradation), it is possible that autosomal targets produce amplicons while amplification of Y targets is unsuccessful, which could be reflected in particular when processing samples with low DNA quantity and quality. When qPCR analyses using two different qPCR kits amplifying autosomal and Y-chromosomal targets were performed on 80 bone samples up to 50 years old, a lower amplification success rate was observed on Y targets than autosomal targets^52^. Because two targets differing in length were amplified on autosomes and the Y chromosome, it was possible to calculate the degradation index, and a higher level of DNA degradation was determined for the Y chromosome, reflecting more efficient amplification of autosomal than Y-chromosomal STRs^52^. Differences in amplification efficiency were also observed between autosomal and Y-chromosomal single nucleotide polymorphisms (SNP) of the Precision ID Identity kit (TFS) used in massive parallel sequencing of forensic samples, showing lower sensitivity of the Y-specific identity SNP markers^53–56^. The structure of the Y chromosome differs from autosomes in more repetitive sequences^57^, making the Y chromosome more susceptible to degradation, resulting in lower amplification success of Y-chromosomal markers compared to autosomal markers.

Another problem in the qPCR test was observed in samples in which a high concentration of autosomal DNA was obtained and very low measures of Y targets were detected (usually below the detection limit of the PowerQuant qPCR kit). This happened in all samples labeled in green in SM 1 (except two low-template samples), and when the Y-STR typing test was performed Y-STR amplicons were not produced in any of those samples, indicating possible detection of contamination. Even in some ENCs (ENC 2, 11 and 12, see SM 2), the Y qPCR target was detected with quantities below the detection limit of the PowerQuant kit and no autosomal STRs were amplified, indicating no serious contamination issue and proving that in bone extracts with high autosomal PowerQuant measures and very low Y-target measures no endogenous male DNA from bones was present.

When highly degraded DNA was used for sex identification through the Y-STR amplification test, only short STRs produced amplicons and longer ones failed (see SM 1 and SM 4, samples labeled in yellow). However, male DNA was confirmed through amplification of shorter targets that were used in the qPCR Y-target test and amelogenin test.

Limitations of sex informative tests used in routine forensic analyses are mainly connected with low-quantity and low-quality samples. However, when high DNA-yielding bones are tested with the amelogenin test it can happened that the Y target does not amplify due to mutations or the presence of null alleles^58–61^, and analyses of additional sex informative markers can solve this problem.

When the amount of DNA did not limit the success of amplification of sex informative markers, a combination of up to three sex informative tests—the qPCR Y-target test, amelogenin test, and Y-STR amplification test—provided reliable sex identification in 78 out of 83 skeletons analyzed.

Poor DNA preservation can prevent determination of sex by genetic methods. The bones of immature individuals have low tensile and compressive strength, are badly mineralized, are highly porous, have high organic content, and have a large exterior accessible for chemical reaction, and are consequently more susceptible to decay^62–69^. Poor preservation of subadult remains is likely to result in the full decomposition of certain bones or the entire skeleton. Consequently, subadults are frequently underrepresented in excavated human remains, and young subadults are especially rare in excavation sites^66,70–73^. When preserved, subadult skeletal remains are rarely maintained sufficiently for ancient DNA extraction^74^. However, at the Ljubljana–Polje archeological site more subadults than adults were excavated and more than half of the subadults still had petrous bones preserved, presumably because this is a modern-era cemetery dating to 17th to 19th century, which in ancient investigations does not represent a long period of exposure of skeletons to unfavorable and harmful environmental conditions. It has been shown that not all bones have equally suitability for genetic analysis because of differences in DNA preservation^24,75–80^, particularly in ancient skeletons, which are subject to decay for a greater time. Numerous studies have shown that the petrous part of the temporal bone yields the most DNA in ancient skeletal remains because its bone tissue is very hard and dense, and therefore more resistant to decay^24,33,34,36,42,81–83^. When the most appropriate bone type sampling strategy was studied for ancient subadult skeletons, it was found that, among the various skeletal elements analyzed, only petrous bones yielded a sufficient quantity of DNA for successful STR genetic typing^84^. To acquire the best-preserved DNA from the skeletons that were studied, we selected the petrous bones whenever possible. Only in three subadults was the petrous bone not preserved, and femurs were sampled instead. Less than 0.5 ng of DNA per gram of femur was obtained from them, and very low DNA yield prevented successful sex assessment in all three femurs analyzed. Out of 80 petrous bones studied, only two failed successful sex identification, indicating highly preserved DNA in petrous bones. Sex was successfully determined in 94% of subadults studied with no differences regardless of age, and the main difference was observed regarding the type of skeletal element sampled for DNA analyses. Namely, all femurs failed in successful genetic sexing. Various studies performed on ancient subadults show different bone preservation in accordance with their age, so that the human remains of the youngest subadults (below age 3) are less well preserved than the bones of older subadults concerning different bone types of the human body^66,70^. However, despite the fact that almost 40% of the subadults studied here were under 1 year old (22% of the skeletons even 30 to 40 weeks old in utero), we managed to determine their sex using genetic methods. The reason for successful sex identification regardless of the age of the subadults studied can be found in the selection of the skeletal element type. The otic capsule of the petrous bone was used for obtaining the bone powder from all but three subadults studied. The otic capsule has unique characteristics that are the result of specific embryonic development that is not characteristic for other bone types. Namely, the development of the otic capsule is already completed in the embryonic stage. The otic capsule is formed when the mesenchyme lining the inner ear differentiates into cartilage^85^. Ossification of the cartilage that was previously formed starts by the 16th week of gestation, when the membranous labyrinth attains its full size. Ossification starts in 14 centers that coalesce to create a capsule that encircles the perilymphatic space and membranous labyrinth^86^. Just prior to birth, ossification is complete^86^, and the otic capsule of newborns exhibits high density, limited vascularization, and absence of bone remodeling^35^, features that result in minimal post-mortem decay of the DNA due to bacterial or other causes^34^, resulting in obtaining a high yield of endogenous DNA even from ancient newborns’ petrous bones.

Sampling of petrous bones in adult skeletons is difficult and destructive^87^ because the osseous labyrinth, which yields the highest endogenous DNA levels, is located on the inside of the petrous bone, and accessing the labyrinth causes structural impairment of the cranial base or vault. Because subadult skeletons were examined in this study, there were no problems with sampling the petrous bones. The cranial sutures were not closed, and in individuals younger than 1 year the squamotympanic and petromastoid parts of the temporal bone were not even fused^88^.

According to almost full profiles obtained using autosomal and Y-chromosomal STR typing kits, we assume that not only the sex assessment but also identification of subadults studied would be possible if reference samples were available. However, reference samples in ancient skeletons are very rarely accessible (some historically important persons have been identified through comparison to living distant relatives;^89,90^ in contrast to forensic missing person identification cases involving skeletal remains, in which reference samples are delivered to the laboratory in almost all routine cases. However, when there are no clues regarding who the missing person might be, the genetic profile obtained from skeletal remains is entered into the national missing person genetic database.

## Conclusions

Through the qPCR PowerQuant Y-target amplification test, amelogenin test, and Y-STR amplification test, sex was successfully determined for all but five subadult skeletons, which yielded the lowest amounts of DNA. For 35 skeletons male sex was identified, and in 43 subadults female sex was established. In all but three skeletons for which femurs were processed, petrous bones were used for molecular genetic sex assessment. Excellent preservation of petrous bones made it possible to determine sex in almost all subadults studied, which, in addition to the numerous published aDNA studies^33,34,42,81,83,91^ and forensic DNA studies^35,44,77,79,92–94^, confirms the exceptional preservation of DNA in petrous bones. Our study shows that genetic sexing using the DNA approach is very efficient in sex identification of subadult skeletons for which sex is anthropologically undetermined and can be used regardless of the age of the deceased child. It can be used in infanticide when only skeletal remains of the newborn are found because sex identification was shown to be successful even in ancient skeletons that belonged to stillborn babies or premature babies with a gestational age of 30–40 weeks. Because studies performed on subadult skeletal remains in forensic investigations^1,95^ and historical or ancient investigations^96–98^ are rare, this study’s results may assist forensic experts when there is a need to determine the sex of badly preserved subadult skeletal remains in routine forensic identification cases. It is essential to take into account that the sampling of petrous bones whenever possible is mandatory for successful DNA analyses of aged subadult skeletal remains.

### Supplementary Information

Supplementary Tables.

## Data Availability

The datasets generated during and/or analysed during the current study are available from the corresponding author on reasonable request.
